# Hyaluronan and LYVE-1 and allograft function in lung transplantation recipients

**DOI:** 10.1038/s41598-019-45309-6

**Published:** 2019-06-21

**Authors:** Andrew M. Courtwright, Anthony M. Lamattina, Pierce H. Louis, Anil J. Trindade, Patrick Burkett, Jewel Imani, Shikshya Shrestha, Miguel Divo, Steve Keller, Ivan O. Rosas, Hilary J. Goldberg, Souheil El-Chemaly

**Affiliations:** 1 Hospital of the University of Pennsylvania, Philadelphia, PA United States; 2 Brigham and Women’s Hospital, Boston, MA United States

**Keywords:** Prognostic markers, Diagnostic markers, Outcomes research

## Abstract

Hyaluronan (HA) is associated with innate immune response activation and may be a marker of allograft dysfunction in lung transplant recipients. This was a prospective, single center study comparing levels of bronchioalveolar lavage (BAL) and serum HA and the HA immobilizer LYVE-1 in lung transplant recipients with and without acute cellular rejection (ACR). Chronic lung allograft dysfunction (CLAD)-free survival was also evaluated based on HA and LYVE-1 levels. 78 recipients were enrolled with a total of 115 diagnostic biopsies and 1.5 years of median follow-up. Serum HA was correlated with BAL HA (r = 0.25, p = 0.01) and with serum LYVE-1 (r = 0.32, p = 0.002). There was significant variation in HA and LYVE-1 over time, regardless of ACR status. Levels of serum HA (median 74.7 vs 82.7, p = 0.69), BAL HA (median 149.4 vs 134.5, p = 0.39), and LYVE-1 (mean 190.2 vs 183.8, p = 0.72) were not associated with ACR. CLAD-free survival was not different in recipients with any episode of elevated serum HA (HR = 1.5, 95% CI = 0.3–7.7, p = 0.61) or BAL HA (HR = 0.94, 95% CI = 0.2–3.6, p = 0.93). These results did not differ when stratified by bilateral transplant status. In this small cohort, serum HA, BAL HA, and LYVE-1 levels are not associated with ACR or CLAD-free survival in lung transplant recipients.

## Introduction

Although lung transplantation can be a life-saving and quality-of-life-improving intervention in individuals with advanced lung disease, subsequent allograft dysfunction remains common. More than a third of recipients will experience an episode of acute cellular rejection (ACR) in the first year after transplant and almost 50% will develop chronic lung allograft dysfunction (CLAD) by 5 years after transplant^1,2^. By 10 years after transplant, 90% of recipients will have died or developed CLAD^3^. Monitoring for ACR is limited to tracking pulmonary function tests (PFTs) over time in combination with surveillance or for-cause bronchoscopy with biopsy. Because of the potential risks associated with biopsy and the limited sensitivity of PFTs for subclinical rejection, there have been ongoing attempts to identify biomarkers of ACR^4^. Similarly, because the diagnosis of CLAD relies on trends in serial PFT measurements, there is a need to identify biomarkers that identify patients at high risk for CLAD prior to changes in PFT^5^.

Hyaluronan (HA) is a ubiquitously distributed extracellular matrix glycosaminoglycan, which exists physiologically as a high-molecular-weight polymer but undergoes extensive fragmentation and molecular modifications in response to tissue injury^6–10^. HA has been previously associated with lung injury and repair through multiple pathways^6,7,11^. Lymphatic vessels facilitate the uptake of HA fragments through the transmembrane receptor lymphatic vessel endothelial hyaluronan receptor 1 (LYVE-1) on the surface of lymphatic endothelial cells^12,13^. Induction of lymphangiogenesis alleviates acute lung allograft rejection and is associated with decreased low molecular weight (LMW)-HA graft content^14,15^. Moreover, LYVE-1 interactions with different MW-HA can differentially impact cell function and alter immune cell trafficking^16^. HA uptake and binding to lymphatic endothelial cells requires LYVE-1 receptor multimerization^17^. Inflammatory stimuli can lead to decreased LYVE-1 surface expression^18^. Furthermore, matrix metalloproteinases have been shown to cleave LYVE-1 from the cell surface, leading to a soluble form that can be found in the periphery^19^. For example, serum LYVE-1 appears to be elevated in lower respiratory tract infections^20^ and decreased in metastatic lung cancer^21^. Therefore, HA and LYVE-1 are potential biomarkers of lung inflammation.

Small prospective cohort studies from the 1990s, ranging from 10–57 lung transplant recipients, found that elevated serum and BAL HA were associated with ACR but larger contemporary studies are lacking^22–24^. Similarly, although elevated levels of HA have been found in recipients who have developed the bronchiolitis obliterans (BOS) subtype of CLAD, there are no data on whether elevated HA levels are associated with risk for developing CLAD^25^. The objective of this study was to assess the relationship between serum and BAL HA and serum LYVE-1 and ACR and CLAD-free survival. We hypothesized that HA and LYVE-1 would be elevated during episodes of ACR and that having an episode of elevated HA would be a risk factor for worse CLAD-free survival.

## Methods

### Study cohort

This was a prospective single center study from 7/1/2015 to 12/31/2017. Informed consent was obtained from all subjects, all experimental protocols were approved by the Institutional Review Board at Brigham and Women’s Hospital Serum, and all methods were carried out in accordance with relevant guidelines and regulations.

BAL samples were collected from consenting subjects at the time of routine surveillance bronchoscopy. We used Hyaluronan Quantikine enzyme-linked immunosorbent assay (ELISA) kits (R & D Systems, Cat # DHYAL0) and LYVE-1 DuoSet ELISA kits (R & D Systems, Cat # DY2089) to measure HA in the sera (8-fold dilution) and BAL supernatant (8-fold dilution) and LYVE-1 in the sera (1000-fold dilution) of lung-transplanted patients. Samples and standard curves were run in duplicate, with some samples run in subsequent plates on different days to confirm reproducibility of measurements. To normalize HA concentrations in the BAL samples, protein A280 concentrations (mg/mL) of each sample were measured via a NanoDrop 2000c instrument (Thermo Fisher Scientific), and protein-corrected BAL values were calculated by dividing raw HA values by the corresponding A280 protein concentrations.

### Assay variability

To measure intra-assay variability, the coefficient of variation (CV) percentage was calculated for each sample by dividing the standard deviation of the sample’s duplicate concentrations on a given plate by its mean concentration and multiplying by 100. CV % was averaged per plate and mean intra-assay CV % and standard deviation summary values were calculated per ELISA by averaging over per-plate CV means. For inter-assay variability, some samples were run twice per ELISA on assays that were performed on different days. CV percentages were calculated for each of these samples by dividing the standard deviation of the concentrations from each subsequent plate by the respective mean concentration and multiplying the result by 100. Inter-assay CV % mean and standard deviation were measured for each ELISA over the CV values of the samples run multiple times.

### Outcomes

The two primary study outcomes were ACR on routine surveillance bronchoscopy and CLAD-free survival. Transbronchial biopsies were excluded from the analysis if they were non-diagnostic according to international standards^26^. ACR was defined as the presence of Grade A1 or higher acute rejection. The presence of small airway inflammation (Grade B1R or higher) was recorded for separate analysis. CLAD was characterized according to consensus definitions^27^.

### Statistical analysis

All analyses were performed using Stata (Version 15.1, Stata Corp, College Station, Texas). Serum and BAL HA levels were non-normally distributed and compared using a Spearman’s rank correlation coefficient test. Serum LYVE-1 was normally distributed. Changes in serum and BAL HA and in LYVE-1 were compared in the same patient at the time of an episode of ACR and at a time without ACR using a Wilcoxon rank sum test. The relationship between ACR and concurrent serum HA, BAL HA, and LYVE-1 levels—treated both as continuous variables and as categorical variables (highest quartile of HA or LYVE-1 levels)—were compared using Wilcoxon rank sum tests (or Student’s t test for LYVE-1) and Fisher Exact tests, respectively. The relationship between CLAD-free survival and any episode of elevated serum and BAL HA levels—defined as a HA level in the highest quartile of HA levels—was assessed using a Cox proportional hazard model. The proportional hazard assumption was confirmed using Schoenfeld residuals.

We conducted three sensitivities analyses using the same outcomes. First, we log 10 transformed the HA levels as previously reported^28^. Second, we restricted the analysis to individuals with bilateral lung transplant to account for changes in HA and LYVE-1 from the native lung in single lung recipients. Third, in order to account for possible confounding by acute transplant-related inflammation, we restricted our analysis to samples collected after 100 days post-transplant.

## Results

A total of 83 subjects were consented and enrolled in the study and a total of 136 biopsies were obtained. Of these, 21 (15.4%) biopsies had insufficient tissue for diagnostic purposes, leaving a total of 115 biopsies and 78 recipients. Of the included biopsies, 102 (88.7%) had concurrent BAL HA samples (24 patients contributed 2 or more samples), 91 (79.1%) had concurrent serum HA (18 patients contributed 2 or more samples), 90 (78.2%) had concurrent serum LYVE-1 (18 patients contributed 2 or more samples), and 80 (69.6%) had both BAL and serum HA samples. Characteristics of the study cohort are listed in Table 1. The median follow-up time was 552 days (interquartile range (IQR) = 335–786) and there were 3 (3.8%) deaths during the study period.

**Table 1:** Study cohort characteristics.

Age (years), mean ± SD	55.9 ± 11.2
Female, n (%)	31 (39.7)
Diagnosis, n (%)	
Interstitial lung disease	42 (53.8)
Chronic obstructive pulmonary disease	16 (20.5)
Cystic fibrosis	11 (14.1)
Other	9 (11.5)
Lung allocation score, median (IQR)	39.6 (34–51.1)
Wait time (days), median (IQR)	162 (45–394)
Bilateral, n (%)	61 (78.2)
Median diagnostic biopsies per recipient	1 (1–2)
Any episode of acute cellular rejection, n (%)	27 (34.6)
Developed chronic lung allograft dysfunction, n (%)	9 (12.5)
Died during study, n (%)	3 (3.8)
Follow-up time (days), median (IQR)	552 (335–786)

Per-plate intra-assay coefficient of variation (CV) means were less than 10% for all ELISA plates run for HA (3.9 ± 2.0%, n = 9) and LYVE-1 (3.8 ± 0.9%, n = 4) measurement. HA ELISA plates featured a mean inter-assay CV mean less than 15% (2.8 ± 3.5%, n = 5), while LYVE-1 plates featured a slightly higher inter-assay CV mean (19.0 ± 11.6%, n = 12).

### Relationship between serum HA, BAL HA, and LYVE-1

**Figure 1:**
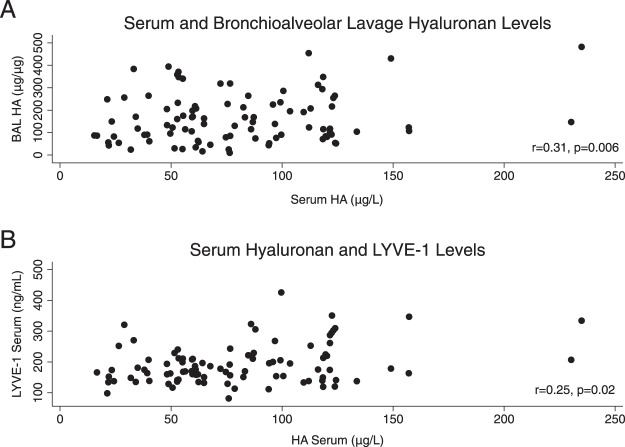
Correlation between (**A**) serum and bronchioalveolar lavage (BAL) hyaluronan (HA) and (**B**) correlation between serum HA and serum lymphatic vessel endothelial hyaluronan receptor 1 (LYVE-1).

**Figure 2:**
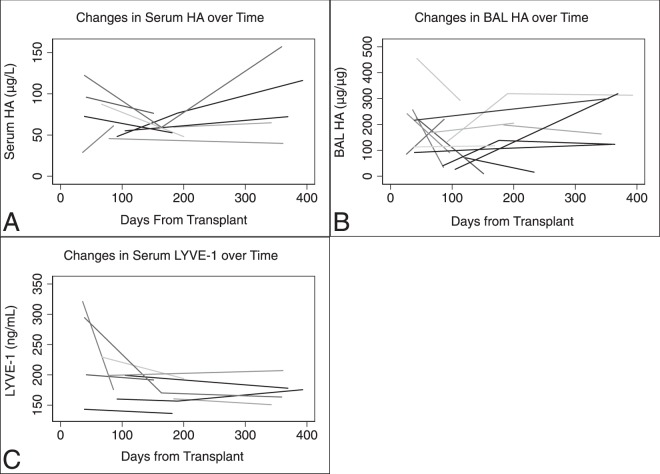
Changes in (**A**) serum and (**B**) bronchioalveolar lavage (BAL) hyaluronan (HA) over time and (**C**) serum lymphatic vessel endothelial hyaluronan receptor 1(LYVE-1) over time, excluding episodes of acute cellular rejection. Each line represents a single patient.

Serum HA was correlated with corrected BAL HA (r = 0.31, p = 0.006) but not uncorrected BAL HA (r = 0.12, p = 0.28) (Fig. 1A). Serum LYVE-1 was not correlated with corrected BAL HA (r = 0.00, p = 0.98) but was correlated with serum HA (r = 0.25, p = 0.02) (Fig. 1B). Among recipients with repeat serum HA levels available and no ACR events (n = 9) the median absolute change in serum HA over time was 28.3 μg/L (IQR = 18.3–39.3) (Fig. 2A–C). Among recipients with repeat LYVE-1 levels available and no ACR events (n = 9) the median absolute change over time was 9.7 ng/mL (IQR = 7.4–28.4). Among recipients with repeat BAL HA levels available and no ACR events (n = 14), the median absolute change in BAL HA was 50.2 μg/μg of protein (IQR = 15.6–158.7). The change in BAL HA over time was not more significant than the change in serum HA over time (p = 0.10).

### Serum HA, BAL HA, and LYVE-1 and ACR

**Figure 3:**
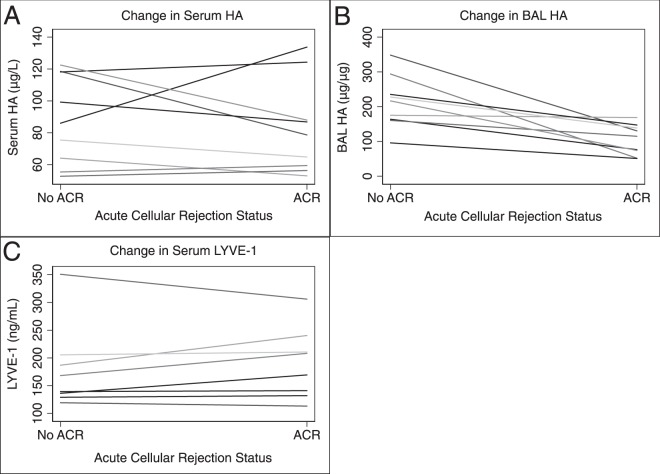
(**A**) Changes in serum for the same patient during an episode of acute cellular rejection (ACR) versus without ACR. Serum HA was not higher during an episode of ACR (median 71.7 vs 87.3, p = 0.83). (**B**) Changes in bronchioalveolar lavage (BAL) hyaluronan (HA) for the same patient during an episode of acute cellular rejection (ACR) versus without ACR. BAL HA was higher in recipients without ACR than during an episode of ACR (median 216.5 vs 114.9, p = 0.003). **(C)** Changes in serum lymphatic vessel endothelial hyaluronan receptor 1 (LVYE-1) for the same patient during an episode of acute cellular rejection (ACR) versus without ACR. Serum LYVE-1 was not higher during an episode of ACR than without ACR (179.5 ± 75.4 vs 190.2 ± 64.2, p = 0.76).

A total of 27 (34.6%) patients had at least one episode of ACR. Comparing changes in HA for the same recipient with and without ACR (n = 9), serum HA was not higher during an episode of ACR than without ACR (71.7, IQR = 57.9–87.4 vs 87.3, IQR = 55.3–118.3, p = 0.83) (Fig. 3A). In contrast, BAL HA was higher in recipients without ACR than in the same recipient during an episode of ACR (216.5, IQR = 163.9–235.3 vs 114.9, IQR = 74.1–138.6, p = 0.003) (Fig. 3B). Finally, LYVE-1 was not higher during an episode of ACR than without ACR (179.5 ± 75.4 vs 190.2 ± 64.2, p = 0.76) (Fig. 3C). Neither serum HA (median 72.6 vs 82.7, p = 0.57), BAL HA (149.4 vs 134.5, p = 0.57), nor LYVE-1 (mean 190.2 ± 66.7 vs 183.8 ± 53.7, p = 0.72) was associated with ACR (Table 2). These results did not change when HA or LYVE-1 were treated as categorical variables (highest quartile) or when considering individuals with elevated HA and reduced LYVE-1 (Table 2). Neither serum HA (99.2, IQR = 64.2–112.5 vs 73.6, IQR = 48.1–116.1, p = 0.20), BAL HA (76.7, IQR = 45.2–224.0 vs 148.9, IQR = 83.4–254.8, p = 0.22), nor LYVE1 (175.9 ± 70.2 vs 190.8 ± 68.1, p = 0.59) was associated with airway (B grade) rejection. These results did not change when A and B grade rejection episodes were combined (data not shown)

**Table 2:** Relationship between serum HA (n = 91), BAL HA (n = 102), and serum LYVE-1 (n = 90) and acute cellular rejection.

	No ACR	ACR	p-value
BAL HA, median IQR	149.4 (79.2–227.7)	134.5 (67.7–158.0)	0.54
Serum HA, median IQR	72.6 (50.5–112.0)	82.7 (57.8–97.1)	0.57
Elevated BAL HA, n (%)	23 (26.7)	3 (18.7)	0.75
Elevated serum HA, n (%)	18 (24.0)	3 (18.7)	0.76
LYVE-1, mean, SD	190.2 ± 66.7	183.8 ± 53.7	0.72
Reduced LYVE-1, n (%)	17 (22.9)	5 (31.2)	0.52
Reduced LYVE-1 and elevated serum HA, n (%)	5 (6.7)	1 (6.2)	1.00
Elevated LYVE-1 and elevated serum HA, n (%)	8 (10.8)	0 (0.0)	0.34
Reduced LYVE-1 and elevated BAL HA, n (%)	6 (8.1)	0 (0.0)	0.59

ACR = acute cellular rejection.

BAL = bronchioalveolar lavage.

HA = Hyaluronan.

LYVE-1 = lymphatic vessel endothelial hyaluronan receptor 1.

IQR = interquartile range.

### Serum and BAL HA and CLAD-free survival

**Figure 4:**
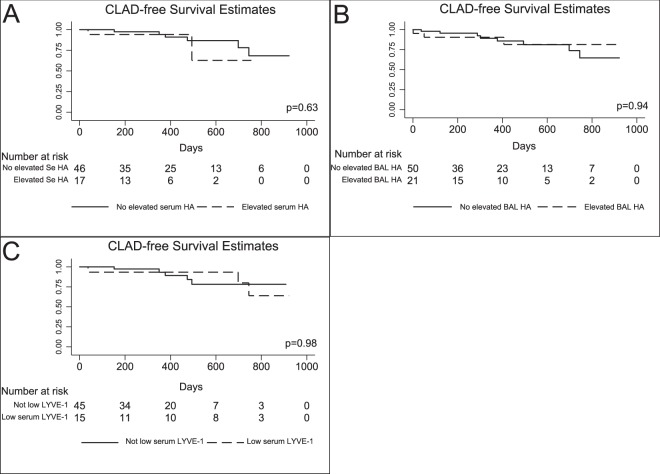
Differences in chronic lung allograft dysfunction (CLAD)-free survival in patients with and without an (**A**) elevated serum (Se) and (**B**) bronchioalveolar lavage (BAL) hyaluronan (HA) level and (**C**) reduced serum LYVE-1.

A total of 9 (12.5%) developed CLAD during study follow-up, all of which were cases of obstructive CLAD. Recipients with any episode of elevated serum HA—defined as a HA level in the highest quartile of HA levels (n = 15 with serum HA >112.4)—did not have worse CLAD-free survival (hazard ratio [HR] = 1.5, 95% confidence interval [CI] = 0.3–7.7, p = 0.61) (Fig. 4A). Elevated BAL HA (n = 22 in highest quartile (with BAL HA >65.8)) was not associated with CLAD-free survival (HR = 0.94, 95% CI = 0.2–3.6, p = 0.93) nor was reduced LYVE-1 (n = 15 in the lowest quartile (with LYVE-1 < 140.4)) (HR = 1.01, 95% CI = 0.23–4.45, p = 0.98) (Fig. 4B,C). Reduced LYVE-1 in combination with elevated serum HA was not associated with CLAD free-survival (HR = 2.89, 95% CI = 0.34–24.56, p = 0.33). Finally, the combination of ACR on biopsy and elevated BAL HA was not associated with CLAD-free survival (HR = 2.8, 95% CI = 0.3–22.7, p = 0.33).

### Sensitivity analyses

Log 10 transforming the HA data did not change our study findings (data not shown). Similarly, restricting the analysis to bilateral transplant recipients—to avoid possible confounding because of native lung HA or LYVE-1 production in single lung recipients—did not alter our study findings (Supplemental Tables 1 and 2 and Supplemental Fig. 1A–C). Finally, we did not find a relationship between late ACR (occurring >100 days post-transplant) and serum HA (p = 0.49), BAL HA (p = 0.99), or LYVE-1 (p = 0.93).

## Discussion

Although HA has been hypothesized to be associated with ACR and CLAD in lung transplant recipients and LYVE-1 mediates HA clearance, our primary findings in this prospective single center cohort study were: (1) serum and BAL HA are modestly correlated; (2) serum HA, BAL HA, and LYVE-1 have significant variability over time and are not associated with ACR; (3) having an episode of elevated serum or BAL HA is not a marker for worse CLAD-free survival; (4) for the same patient BAL HA is lower during an episode of ACR.

HA has long been associated with lung injury and respiratory diseases ranging from emphysema to sarcoidosis to pulmonary fibrosis^29,30^. Given its dual function as extracellular matrix component and modulator of inflammatory response, HA is attractive as a potential biomarker of injury and a potential target for intervention. Similar to prior data in advanced lung disease, we found relatively modest correlations between serum and BAL HA^29^. Median and mean serum HA levels (74.7 μg/L and 78.6 μg/L, respectively) in the cohort were somewhat higher than in other studies in lung transplantation, which range from the 30 s to 60 s^22,23^. Median and mean uncorrected BAL HA levels (37.6 μg/L and 47.5 μg/L, respectively) were in keeping with earlier studies^22,25^. BAL HA levels were, however, significantly higher in our cohort than those reported in a recent abstract, even after accounting for log 10 transformation^28^. Among patients who did not experience a rejection episode, there was still significant variation in sequential BAL and serum HA measurements. For example, 25% of these individuals had a change in BAL HA greater than 159 μg/μg, suggesting significant baseline variability in HA levels even without ACR on biopsy.

In contrast to several small cohort studies in the 1990s, we did not find that increased BAL HA levels were associated with ACR either in general or in the same patient during an episode of ACR versus an episode without ACR. There are several possible explanations for this difference. Because we enrolled significantly more patients, we may have been able to avoid type I error. Alternatively, BAL HA levels were higher in recipients in our cohort who did not have rejection (74.7 μg/L) than in other studies, where levels have been reported between 33–62 μg/L^22,25^. A higher baseline BAL HA may have limited our ability to detect smaller changes that occurred in ACR. In keeping with Riise *et al*.^23^ we did not find a significant relationship between serum HA and ACR.

Interestingly, we found a decline in BAL HA in the same recipient during a period of rejection, although this observation remains hypothesis-generating given the relatively small number of recipients in this subgroup. These findings could be related to the lack of release of HA fragments from the injured tissue to the alveolar milieu or perhaps more likely related to the ELISA itself. Hyaluronic acid binding protein (HABP) is used to measure HA in these ELISA kits. HABP could bind more frequently to high molecular weight HA compared to LMW-HA and therefore could lead to a higher reading of HA concentration. Future analyses identifying both the absolute amount and relative proportion of high and low molecular weight HA fragments in study specimens is necessary to clarify whether Low molecular weight HA (LMWHA) may be a biomarker for ACR.

This is the first study to specifically evaluate whether the HA immobilizer LYVE-1 may serve as a marker of ACR, separately or in conjunction with serum HA. Although serum LYVE-1 has been reported to be elevated during respiratory infections, there are no prior data in lung transplant to compare LYVE-1 levels with our cohort^20^. Even for recipients with elevated HA and low LYVE-1, which may be a group that has reduced HA clearance, there was not an association with ACR or CLAD-free survival.

Given that the inability to clear HA is associated with ACR in murine models, the presence of persistently elevated HA may be a more relevant marker for ACR (and perhaps subsequent CLAD) than a single episode of raised HA^14,25^. In addition, there are data to suggest that the impact of HA on allograft function is partially mediated by HA fragment size^25^. For example, LMWHA appears to promote dendritic cell activation via TLR pathways, which in turn promote alloreactive CD4+ T cell priming^11^.

Regarding the relationship between HA and CLAD, Todd *et al*. previously reported that transplant recipients with BOS had higher levels of serum and BAL HA than those without BOS^25^. BAL HA was not elevated among recipients with a sample available before the onset of BOS, which is consistent with our findings. Todd *et al*.^25^ did not, however, assess whether having an elevated BAL HA (without or without concurrent ACR) was a risk factor for subsequent BOS. There were insufficient numbers of patients with restrictive allograft syndrome (RAS) to characterize whether elevated HA might be associated with this CLAD subtype. As with other efforts to identify BAL cellular, cytokine, and protein constituents to diagnosis or risk-stratify recipients for CLAD, the lack of association between HA, LYVE-1, and CLAD-free survival in our study again suggests that no single biomarker may have sufficient sensitivity in this area^30^.

### Limitations

Although this is one of the largest prospective studies of HA and lung transplant allograft outcomes, there were a limited number of patients with multiple sequential biopsies and serial HA/LYVE-1 levels available for analysis. Thus, while the general lack of association between HA and ACR was powered for moderate-to-large effect sizes, we do not know whether closer monitoring of HA levels over time (potentially in conjunction with LYVE-1 levels) may, for individual patients, reveal associations with ACR or CLAD-free survival. In addition, the NanoDrop method provides an estimation of protein concentration rather than a direct measurement. We also did not collect data on active infections at the time of bronchoscopy, which may have confounded our analysis as active respiratory infections have been associated with elevated HA^22^. Although we intend to collect these data in future studies of biomarkers of ACR where infection may be confounding, we note that it can be difficult to differentiate between acute infection and colonization in the transplant population. Third, because the median follow-up in our cohort was 1.5 years, we do not know whether elevated HA would have been associated with worse CLAD-free survival within a longer follow-up period. It is, however, notable that we did not find a trend toward significance during the current study time. Finally, there were insufficient numbers of high-grade ACR episodes or high-stage CLAD for us to perform a sub-group analysis as to whether HA or LYVE-1 is associated with more severe ACR or CLAD.

## Conclusions

Serum HA and serum LYVE-1 levels are not associated with ACR or CLAD-free survival in lung transplant recipients.

## Supplementary information

Supplemental Information

## Data Availability

The datasets generated and analyzed during the current study are not publically available due to patient confidentiality and privacy protections but may be available in de-identified form the corresponding author on reasonable request.
