# Supplementary material for: Hyaluronan and LYVE-1 and allograft function in lung transplantation recipients
Source: Sci Rep. 2019 Jun 21;9:9003. doi: 10.1038/s41598-019-45309-6 (PMC6588572; doi:10.1038/s41598-019-45309-6)
Supplement: Supplementary file 1 — Supplemental Information [file 41598_2019_45309_MOESM1_ESM.pdf]

Supplemental Data

Title: Hyaluronan and LYVE-1 and allograft function in lung transplantation recipients

Authors: Andrew M. Courtwright[1], Anthony Lamattina[2], Pierce Louis[2], Anil Trindade[2], Patrick Burkett[2], Jewel Imani[2], Shikshya Shrestha[2], Miguel Divo[2], Steve Keller[2], Ivan O. Rosas[2], Hilary J. Goldberg[2], Souheil El-Chemaly[2*]

Author Affiliations:

[1] Hospital of the University of Pennsylvania, Philadelphia, PA

[2] Brigham and Women's Hospital, Boston, MA

***Corresponding author:**

Souheil El-Chemaly, MD, MPH

Thorn Biosciences Research Building

Room 805

Boston, MA 02115

Phone: 617-732-6869 Fax: 617-582-6102

Email: sel-chemaly@bwh.harvard.edu

1) Analyses restricted to bilateral lung transplant recipients only

Serum HA was correlated with corrected BAL HA (r=0.35, p=0.006) and with LYVE-1 (r=0.27, p=0.05). Serum LYVE-1 was not correlated with BAL HA (r=0.02, p=0.87).

Table 1s. Relationship between serum HA (n=64), BAL HA (n=71), and serum LYVE-1 (n=64) and acute cellular rejection, bilateral transplant recipients only.

	No ACR	ACR	p-value
BAL HA, median IQR	169.7 (89.1-251.6)	138.6 (61.4-147.3)	0.23
Serum HA, median IQR	74.7 (48.1-99.6)	87.9 (64.8-112.5)	0.19
Elevated BAL HA, n (%)	16 (26.7)	1 (9.1)	0.27
Elevated serum HA, n (%)	14 (26.4)	2 (18.2)	0.72
LYVE-1, mean, SD	197.4 ± 70.7	191.0 ± 58.6	0.78
Reduced LYVE-1, n(%)	13 (24.5)	5 (31.2)	0.52
Reduced LYVE-1 and elevated serum HA, n (%)	4 (7.5)	0 (0.0)	1.00
Reduced LYVE-1 and elevated BAL HA, n (%)	5 (9.4)	0 (0.0)	0.58

ACR = acute cellular rejection; BAL = bronchioalveolar lavage; HA = Hyaluronan; LYVE-1 = lymphatic vessel endothelial hyaluronan receptor 1; IQR = interquartile range

**Table 2s. Relationship between elevated serum HA, BAL HA, or reduced LYVE-1 and CLAD-free survival, bilateral transplant recipients only.**			
	**HR**	**95% CI**	**p-value**
Elevated BAL HA, n (%)	NA[a]		
Elevated serum HA, n (%)	1.88	0.17-20.67	0.61
Reduced LYVE-1, n(%)	2.72	0.38-19.31	0.32

[a] No CLAD or morality events among recipients with elevated BAL HA

BAL = bronchioalveolar lavage; CI = confidence interval; CLAD = chronic lung allograft dysfunction; HA = Hyaluronan; HR = hazard ratio; LYVE-1 = lymphatic vessel endothelial hyaluronan receptor 1

Supplemental Figure 1. Changes in (A) serum over time, (B) bronchioalveolar lavage (BAL) hyaluronan (HA) over time, and (C) serum lymphatic vessel endothelial hyaluronan receptor 1(LYVE-1) over time, excluding episodes of acute cellular rejection. Each line represents a single patient.

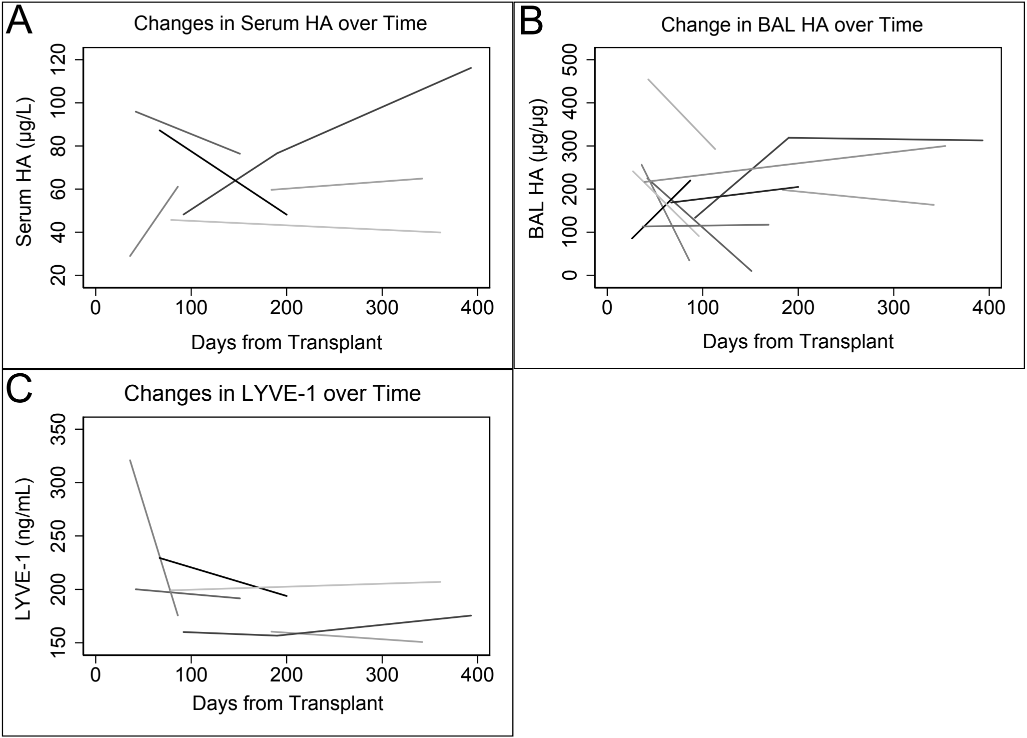

A
Changes in Serum HA over Time
Serum HA (µg/L)
20
40
60
80
100
120
0
100
200
300
400
Days from Transplant
B
Change in BAL HA over Time
BAL HA (µg/µg)
0
100
200
300
400
500
0
100
200
300
400
Days from Transplant
C
Changes in LYVE-1 over Time
LYVE-1 (ng/mL)
150
200
250
300
350
0
100
200
300
400
Days from Transplant

2) Mixed effect model

To assess whether the inclusion of multiple samples by the same subject biased our primary outcome analyses, we used a mixed effects logistic regression model with ACR as the dependent variable and serum HA, BAL HA, and serum LYVE-1, separately, as the independent variable(s), allowing random effects to vary according to subject. For ease of interpretation of the resulting odds ratios and confidence intervals, we 10-fold reduced all independent variables.

Supplemental Table 3. Relationship between serum HA (n=91), BAL HA (n=102), and serum LYVE-1 (n=90) and acute cellular rejection, mixed effects model.

	OR	95% CI	p-value
BAL HA, median IQR	0.99	0.94-1.04	0.67
Serum HA, median IQR	1.04	0.92-1.19	0.49
LYVE-1, mean, SD	0.98	0.90-1.07	0.72

BAL = bronchioalveolar lavage; CI = confidence interval; HA = Hyaluronan; LYVE-1 = lymphatic vessel endothelial hyaluronan receptor 1; OR = odds ratio
